# 2-Methyl­propan-2-aminium 3,4,5,6-tetra­bromo-2-(meth­oxy­carbon­yl)benzoate methanol monosolvate

**DOI:** 10.1107/S1600536811008518

**Published:** 2011-03-12

**Authors:** Jian Li

**Affiliations:** a Department of Chemistry and Chemical Engineering, Weifang University, Weifang 261061, People’s Republic of China

## Abstract

In the crystal structure of the title compound, C_4_H_12_N^+^·C_9_H_3_Br_4_O_4_^−^·CH_4_O, inter­molecular O—H⋯O, N—H⋯O and C—H⋯O hydrogen bonds link the components into columns stacked along the *b* axis. Between the columns, short Br⋯O contacts [3.122 (4) Å] and C—H⋯O hydrogen bonds are observed.

## Related literature

For related structures, see: Li (2011 ▶); Liang (2008 ▶). 
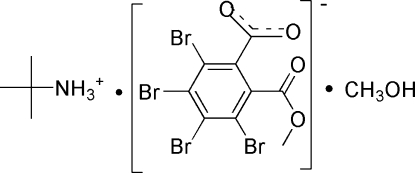


## Experimental

### 

#### Crystal data

C_4_H_12_N^+^·C_9_H_3_Br_4_O_4_^−^·CH_4_O
*M* *_r_* = 600.94
Monoclinic, 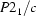

*a* = 12.3832 (11) Å
*b* = 8.4001 (6) Å
*c* = 20.6394 (18) Å
β = 107.316 (1)°
*V* = 2049.6 (3) Å^3^
*Z* = 4
Mo *K*α radiation
μ = 7.88 mm^−1^
*T* = 298 K
0.39 × 0.30 × 0.24 mm

#### Data collection

Bruker SMART CCD area-detector diffractometer
Absorption correction: multi-scan (*SADABS*; Bruker, 1997 ▶) *T*_min_ = 0.149, *T*_max_ = 0.254
9926 measured reflections
3597 independent reflections
2071 reflections with *I* > 2σ(*I*)
*R*_int_ = 0.062

#### Refinement

*R*[*F* ^2^ > 2σ(*F* ^2^)] = 0.041
*wR*(*F* ^2^) = 0.072
*S* = 1.06
3597 reflections
217 parameters
H-atom parameters constrained
Δρ_max_ = 0.45 e Å^−3^
Δρ_min_ = −0.52 e Å^−3^

### 

Data collection: *SMART* (Bruker, 1997 ▶); cell refinement: *SAINT* (Bruker, 1997 ▶); data reduction: *SAINT*; program(s) used to solve structure: *SHELXS97* (Sheldrick, 2008 ▶); program(s) used to refine structure: *SHELXL97* (Sheldrick, 2008 ▶); molecular graphics: *SHELXTL* (Sheldrick, 2008 ▶) and *PLATON* (Spek, 2009 ▶); software used to prepare material for publication: *SHELXTL*.

## Supplementary Material

Crystal structure: contains datablocks global, I. DOI: 10.1107/S1600536811008518/is2668sup1.cif

Structure factors: contains datablocks I. DOI: 10.1107/S1600536811008518/is2668Isup2.hkl

Additional supplementary materials:  crystallographic information; 3D view; checkCIF report

## Figures and Tables

**Table 1:** Hydrogen-bond geometry (Å, °)

*D*—H⋯*A*	*D*—H	H⋯*A*	*D*⋯*A*	*D*—H⋯*A*
N1—H1*A*⋯O4^i^	0.89	1.91	2.775 (6)	162
N1—H1*B*⋯O3	0.89	2.03	2.916 (6)	177
N1—H1*C*⋯O5^ii^	0.89	1.96	2.837 (6)	168
O5—H5⋯O3	0.82	1.91	2.723 (6)	168
C13—H13*C*⋯O2^iii^	0.96	2.58	3.491 (8)	158
C20—H20*B*⋯O2^iv^	0.96	2.57	3.449 (8)	153

Symmetry codes: (i) 
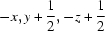
; (ii) 
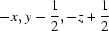
; (iii) 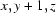; (iv) 
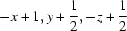
.

**Fig. 1.**
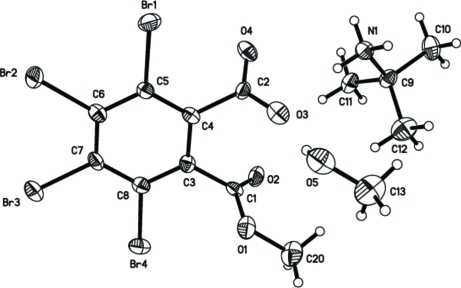
The molecular structure of the title compound with 30% probability ellipsoids.

**Fig. 2.**
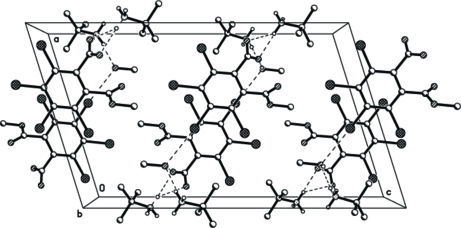
A packing diagram, viewed along the b axis.

## supplementary crystallographic information

### Comment

4,5,6,7-Tetrabromo-2-ethylisoindoline-1,3-dione is an important flame retardant. 2-(Methoxycarbonyl)-3,4,5,6-tetrabromobenzoic acid is the intermediate of the flame retardant.

In this paper, the structure of the title compound is reported (Fig. 1).  The bond lengths and angles agree with those in the similar compounds (Liang, 2008; Li, 2011). The crystal structure is stabilized by N—H···O hydrogen bonds between the 2-methylpropan-2-aminium cation and the 2-(methoxycarbonyl)-3,4,5,6-tetrabromobenzoate anion, and by O—H···O and N—H···O hydrogen bonds between methanol, 2-(methoxycarbonyl)-3,4,5,6-tetrabromobenzoate and 2-methylpropan-2-aminium (Table 1 and Fig. 2).

### Experimental

A mixture of 4,5,6,7-tetrabromoisobenzofuran-1,3-dione (4.64 g, 0.01 mol) and methanol (15 ml) was refluxed for 0.5 h. Ethanamine (0.73 g, 0.01 mol) was then added to the above solution, being mixed round for 10 min at room temperature. The solution was kept at room temperature for 5 d. Natural evaporation gave colourless single crystals of the title compound suitable for X-ray analysis.

### Refinement

H atoms were initially located in a difference map and then refined in a riding model, with C—H = 0.96 Å, N—H = 0.89 Å and O—H = 0.82 Å, and with *U*_iso_(H) = 1.5*U*_eq_(parent atom).

### Crystal data

**Table:**

C_4_H_12_N^+^·C_9_H_3_Br_4_O_4_^−^·CH_4_O	*F*(000) = 1160
*M**_r_* = 600.94	*D*_x_ = 1.947 Mg m^−^^3^
Monoclinic, *P*2_1_/*c*	Mo *K*α radiation, λ = 0.71073 Å
Hall symbol: -P 2ybc	Cell parameters from 1758 reflections
*a* = 12.3832 (11) Å	θ = 2.6–22.7°
*b* = 8.4001 (6) Å	µ = 7.88 mm^−^^1^
*c* = 20.6394 (18) Å	*T* = 298 K
β = 107.316 (1)°	Block, colorless
*V* = 2049.6 (3) Å^3^	0.39 × 0.30 × 0.24 mm
*Z* = 4	

### Data collection

**Table:**

Bruker SMART CCD area-detector diffractometer	3597 independent reflections
Radiation source: fine-focus sealed tube	2071 reflections with *I* > 2σ(*I*)
graphite	*R*_int_ = 0.062
φ and ω scans	θ_max_ = 25.0°, θ_min_ = 2.6°
Absorption correction: multi-scan (*SADABS*; Bruker, 1997)	*h* = −14→14
*T*_min_ = 0.149, *T*_max_ = 0.254	*k* = −9→6
9926 measured reflections	*l* = −24→24

### Refinement

**Table:**

Refinement on *F*^2^	Primary atom site location: structure-invariant direct methods
Least-squares matrix: full	Secondary atom site location: difference Fourier map
*R*[*F*^2^ > 2σ(*F*^2^)] = 0.041	Hydrogen site location: inferred from neighbouring sites
*wR*(*F*^2^) = 0.072	H-atom parameters constrained
*S* = 1.06	*w* = 1/[σ^2^(*F*_o_^2^) + (0.0138*P*)^2^] where *P* = (*F*_o_^2^ + 2*F*_c_^2^)/3
3597 reflections	(Δ/σ)_max_ < 0.001
217 parameters	Δρ_max_ = 0.45 e Å^−^^3^
0 restraints	Δρ_min_ = −0.52 e Å^−^^3^

### Special details

**Table:**

Geometry. All e.s.d.'s (except the e.s.d. in the dihedral angle between two l.s. planes) are estimated using the full covariance matrix. The cell e.s.d.'s are taken into account individually in the estimation of e.s.d.'s in distances, angles and torsion angles; correlations between e.s.d.'s in cell parameters are only used when they are defined by crystal symmetry. An approximate (isotropic) treatment of cell e.s.d.'s is used for estimating e.s.d.'s involving l.s. planes.
Refinement. Refinement of *F*^2^ against ALL reflections. The weighted *R*-factor *wR* and goodness of fit *S* are based on *F*^2^, conventional *R*-factors *R* are based on *F*, with *F* set to zero for negative *F*^2^. The threshold expression of *F*^2^ > σ(*F*^2^) is used only for calculating *R*-factors(gt) *etc*. and is not relevant to the choice of reflections for refinement. *R*-factors based on *F*^2^ are statistically about twice as large as those based on *F*, and *R*- factors based on ALL data will be even larger.

### Fractional atomic coordinates and isotropic or equivalent isotropic displacement parameters (Å^2^)

**Table:**

	*x*	*y*	*z*	*U*_iso_*/*U*_eq_	
Br1	0.11686 (5)	0.40889 (8)	0.47788 (3)	0.0564 (2)	
Br2	0.33036 (5)	0.29201 (9)	0.60590 (3)	0.0532 (2)	
Br3	0.57304 (5)	0.20168 (9)	0.58025 (3)	0.0570 (2)	
Br4	0.59859 (5)	0.23055 (9)	0.42537 (4)	0.0631 (2)	
N1	−0.0325 (3)	0.4861 (5)	0.1881 (2)	0.0428 (13)	
H1A	−0.0599	0.5833	0.1761	0.064*	
H1B	0.0293	0.4928	0.2236	0.064*	
H1C	−0.0844	0.4276	0.1991	0.064*	
O1	0.4384 (3)	0.4674 (5)	0.31058 (19)	0.0504 (11)	
O2	0.3379 (3)	0.2489 (5)	0.2725 (2)	0.0465 (11)	
O3	0.1746 (3)	0.5145 (5)	0.3009 (2)	0.0463 (11)	
O4	0.0805 (3)	0.3093 (5)	0.32508 (19)	0.0477 (11)	
O5	0.2206 (3)	0.8283 (5)	0.2893 (2)	0.0663 (13)	
H5	0.2158	0.7314	0.2923	0.100*	
C1	0.3789 (4)	0.3409 (7)	0.3164 (3)	0.0317 (15)	
C2	0.1642 (5)	0.3974 (8)	0.3359 (3)	0.0337 (14)	
C3	0.3692 (4)	0.3273 (6)	0.3878 (3)	0.0329 (14)	
C4	0.2644 (4)	0.3585 (6)	0.3973 (3)	0.0291 (14)	
C5	0.2554 (4)	0.3503 (6)	0.4636 (3)	0.0326 (15)	
C6	0.3464 (4)	0.3065 (6)	0.5185 (3)	0.0360 (14)	
C7	0.4492 (4)	0.2690 (7)	0.5079 (3)	0.0374 (15)	
C8	0.4596 (4)	0.2806 (7)	0.4422 (3)	0.0378 (15)	
C9	−0.0030 (5)	0.4086 (7)	0.1290 (3)	0.0405 (15)	
C10	−0.1117 (5)	0.4006 (8)	0.0699 (3)	0.070 (2)	
H10A	−0.1402	0.5063	0.0581	0.105*	
H10B	−0.1670	0.3387	0.0829	0.105*	
H10C	−0.0962	0.3518	0.0315	0.105*	
C11	0.0415 (5)	0.2446 (7)	0.1526 (3)	0.0512 (18)	
H11A	−0.0166	0.1836	0.1630	0.077*	
H11B	0.1056	0.2536	0.1924	0.077*	
H11C	0.0640	0.1924	0.1172	0.077*	
C12	0.0869 (5)	0.5111 (7)	0.1136 (3)	0.068 (2)	
H12A	0.1526	0.5147	0.1527	0.102*	
H12B	0.0581	0.6169	0.1025	0.102*	
H12C	0.1071	0.4666	0.0760	0.102*	
C13	0.2318 (6)	0.8673 (8)	0.2255 (4)	0.081 (2)	
H13A	0.1615	0.8480	0.1911	0.122*	
H13B	0.2900	0.8029	0.2168	0.122*	
H13C	0.2517	0.9777	0.2250	0.122*	
C20	0.4511 (5)	0.4985 (8)	0.2440 (3)	0.063 (2)	
H20A	0.4884	0.4101	0.2305	0.094*	
H20B	0.4953	0.5932	0.2458	0.094*	
H20C	0.3778	0.5129	0.2117	0.094*	

### Atomic displacement parameters (Å^2^)

**Table:**

	*U*^11^	*U*^22^	*U*^33^	*U*^12^	*U*^13^	*U*^23^
Br1	0.0443 (4)	0.0832 (6)	0.0473 (4)	0.0174 (4)	0.0219 (3)	0.0091 (4)
Br2	0.0511 (4)	0.0766 (5)	0.0321 (4)	−0.0041 (4)	0.0125 (3)	0.0065 (4)
Br3	0.0408 (4)	0.0761 (5)	0.0448 (4)	0.0081 (4)	−0.0018 (3)	0.0065 (4)
Br4	0.0361 (4)	0.0945 (6)	0.0611 (5)	0.0116 (4)	0.0181 (3)	0.0003 (4)
N1	0.039 (3)	0.048 (3)	0.039 (3)	0.005 (3)	0.008 (2)	0.000 (3)
O1	0.055 (3)	0.059 (3)	0.041 (3)	−0.028 (2)	0.020 (2)	−0.006 (2)
O2	0.057 (3)	0.045 (3)	0.040 (3)	−0.009 (2)	0.017 (2)	−0.014 (2)
O3	0.049 (2)	0.038 (3)	0.047 (3)	−0.001 (2)	0.008 (2)	0.019 (2)
O4	0.037 (2)	0.048 (3)	0.051 (3)	−0.013 (2)	0.003 (2)	0.004 (2)
O5	0.060 (3)	0.047 (3)	0.087 (4)	0.002 (2)	0.014 (3)	0.004 (3)
C1	0.022 (3)	0.020 (4)	0.048 (4)	0.002 (3)	0.001 (3)	−0.007 (3)
C2	0.040 (4)	0.034 (4)	0.028 (4)	0.003 (3)	0.010 (3)	−0.006 (3)
C3	0.036 (3)	0.026 (4)	0.035 (4)	−0.007 (3)	0.009 (3)	−0.005 (3)
C4	0.026 (3)	0.019 (3)	0.039 (4)	−0.005 (2)	0.006 (3)	0.005 (3)
C5	0.030 (3)	0.035 (4)	0.032 (4)	−0.001 (3)	0.008 (3)	0.006 (3)
C6	0.032 (3)	0.033 (4)	0.039 (4)	−0.004 (3)	0.005 (3)	0.006 (3)
C7	0.031 (3)	0.039 (4)	0.033 (4)	0.005 (3)	−0.004 (3)	0.000 (3)
C8	0.031 (3)	0.041 (4)	0.041 (4)	−0.002 (3)	0.009 (3)	0.001 (3)
C9	0.048 (4)	0.038 (4)	0.037 (4)	0.009 (3)	0.014 (3)	0.000 (3)
C10	0.065 (4)	0.100 (6)	0.032 (4)	0.005 (4)	−0.004 (4)	−0.002 (4)
C11	0.063 (4)	0.049 (5)	0.044 (4)	0.004 (4)	0.019 (3)	−0.009 (3)
C12	0.082 (5)	0.060 (5)	0.074 (5)	−0.004 (4)	0.043 (4)	0.008 (4)
C13	0.102 (6)	0.056 (6)	0.100 (7)	−0.015 (4)	0.051 (5)	−0.001 (5)
C20	0.076 (4)	0.071 (5)	0.050 (5)	−0.023 (4)	0.033 (4)	0.001 (4)

### Geometric parameters (Å, °)

**Table:**

Br1—C5	1.891 (5)	C6—C7	1.390 (7)
Br2—C6	1.878 (5)	C7—C8	1.403 (7)
Br3—C7	1.881 (5)	C9—C11	1.509 (7)
Br4—C8	1.901 (5)	C9—C12	1.514 (7)
N1—C9	1.520 (6)	C9—C10	1.526 (7)
N1—H1A	0.8900	C10—H10A	0.9600
N1—H1B	0.8900	C10—H10B	0.9600
N1—H1C	0.8900	C10—H10C	0.9600
O1—C1	1.318 (6)	C11—H11A	0.9600
O1—C20	1.452 (6)	C11—H11B	0.9600
O2—C1	1.185 (6)	C11—H11C	0.9600
O3—C2	1.250 (6)	C12—H12A	0.9600
O4—C2	1.239 (6)	C12—H12B	0.9600
O5—C13	1.402 (7)	C12—H12C	0.9600
O5—H5	0.8200	C13—H13A	0.9600
C1—C3	1.517 (7)	C13—H13B	0.9600
C2—C4	1.521 (7)	C13—H13C	0.9600
C3—C8	1.386 (7)	C20—H20A	0.9600
C3—C4	1.394 (6)	C20—H20B	0.9600
C4—C5	1.407 (7)	C20—H20C	0.9600
C5—C6	1.389 (6)		
			
C9—N1—H1A	109.5	C12—C9—N1	107.0 (5)
C9—N1—H1B	109.5	C11—C9—C10	111.6 (5)
H1A—N1—H1B	109.5	C12—C9—C10	112.7 (5)
C9—N1—H1C	109.5	N1—C9—C10	107.1 (4)
H1A—N1—H1C	109.5	C9—C10—H10A	109.5
H1B—N1—H1C	109.5	C9—C10—H10B	109.5
C1—O1—C20	116.9 (5)	H10A—C10—H10B	109.5
C13—O5—H5	109.5	C9—C10—H10C	109.5
O2—C1—O1	125.4 (6)	H10A—C10—H10C	109.5
O2—C1—C3	123.6 (5)	H10B—C10—H10C	109.5
O1—C1—C3	111.0 (5)	C9—C11—H11A	109.5
O4—C2—O3	126.2 (5)	C9—C11—H11B	109.5
O4—C2—C4	116.9 (5)	H11A—C11—H11B	109.5
O3—C2—C4	116.8 (5)	C9—C11—H11C	109.5
C8—C3—C4	120.1 (5)	H11A—C11—H11C	109.5
C8—C3—C1	122.0 (5)	H11B—C11—H11C	109.5
C4—C3—C1	117.9 (5)	C9—C12—H12A	109.5
C3—C4—C5	118.3 (5)	C9—C12—H12B	109.5
C3—C4—C2	119.1 (5)	H12A—C12—H12B	109.5
C5—C4—C2	122.6 (5)	C9—C12—H12C	109.5
C6—C5—C4	121.8 (5)	H12A—C12—H12C	109.5
C6—C5—Br1	119.7 (4)	H12B—C12—H12C	109.5
C4—C5—Br1	118.5 (4)	O5—C13—H13A	109.5
C5—C6—C7	119.4 (5)	O5—C13—H13B	109.5
C5—C6—Br2	120.6 (4)	H13A—C13—H13B	109.5
C7—C6—Br2	120.0 (4)	O5—C13—H13C	109.5
C6—C7—C8	119.2 (5)	H13A—C13—H13C	109.5
C6—C7—Br3	120.8 (4)	H13B—C13—H13C	109.5
C8—C7—Br3	120.0 (4)	O1—C20—H20A	109.5
C3—C8—C7	121.2 (5)	O1—C20—H20B	109.5
C3—C8—Br4	118.1 (4)	H20A—C20—H20B	109.5
C7—C8—Br4	120.6 (4)	O1—C20—H20C	109.5
C11—C9—C12	111.5 (5)	H20A—C20—H20C	109.5
C11—C9—N1	106.6 (4)	H20B—C20—H20C	109.5
			
C20—O1—C1—O2	2.2 (8)	C2—C4—C5—Br1	−5.9 (7)
C20—O1—C1—C3	−177.8 (4)	C4—C5—C6—C7	−0.3 (8)
O2—C1—C3—C8	106.6 (7)	Br1—C5—C6—C7	−177.8 (4)
O1—C1—C3—C8	−73.4 (6)	C4—C5—C6—Br2	−178.5 (4)
O2—C1—C3—C4	−71.4 (7)	Br1—C5—C6—Br2	4.0 (6)
O1—C1—C3—C4	108.6 (5)	C5—C6—C7—C8	1.8 (8)
C8—C3—C4—C5	3.7 (8)	Br2—C6—C7—C8	180.0 (4)
C1—C3—C4—C5	−178.3 (5)	C5—C6—C7—Br3	−177.7 (4)
C8—C3—C4—C2	−175.4 (5)	Br2—C6—C7—Br3	0.5 (7)
C1—C3—C4—C2	2.6 (7)	C4—C3—C8—C7	−2.2 (8)
O4—C2—C4—C3	121.4 (6)	C1—C3—C8—C7	179.8 (5)
O3—C2—C4—C3	−57.9 (7)	C4—C3—C8—Br4	177.3 (4)
O4—C2—C4—C5	−57.7 (7)	C1—C3—C8—Br4	−0.7 (7)
O3—C2—C4—C5	123.0 (6)	C6—C7—C8—C3	−0.5 (9)
C3—C4—C5—C6	−2.5 (8)	Br3—C7—C8—C3	178.9 (4)
C2—C4—C5—C6	176.6 (5)	C6—C7—C8—Br4	180.0 (4)
C3—C4—C5—Br1	175.0 (4)	Br3—C7—C8—Br4	−0.6 (7)

### Hydrogen-bond geometry (Å, °)

**Table:**

*D*—H···*A*	*D*—H	H···*A*	*D*···*A*	*D*—H···*A*
N1—H1A···O4^i^	0.89	1.91	2.775 (6)	162
N1—H1B···O3	0.89	2.03	2.916 (6)	177
N1—H1C···O5^ii^	0.89	1.96	2.837 (6)	168
O5—H5···O3	0.82	1.91	2.723 (6)	168
C13—H13C···O2^iii^	0.96	2.58	3.491 (8)	158
C20—H20B···O2^iv^	0.96	2.57	3.449 (8)	153

Symmetry codes: (i) −*x*, *y*+1/2, −*z*+1/2; (ii) −*x*, *y*−1/2, −*z*+1/2; (iii) *x*, *y*+1, *z*; (iv) −*x*+1, *y*+1/2, −*z*+1/2.
